# Retroperitoneum ganglioneuroma: imaging features and surgical outcomes of 35 cases at a Chinese Institution

**DOI:** 10.1186/s12880-021-00643-y

**Published:** 2021-07-22

**Authors:** Qian-Wen Zhang, Tao Song, Pan-Pan Yang, Qiang Hao

**Affiliations:** Department of Radiology, Changhai Hospital, The Navy Medical University (Second Military Medical University), 168 Changhai Road, Shanghai, China

**Keywords:** Ganglioneuroma, Retroperitoneum, Computed tomography, Magnetic resonance imaging, Surgery

## Abstract

**Background:**

The preoperative evaluation is crucial for diagnosis and surgical plan of retroperitoneum ganglioneuroma (GN). In this study, we reviewed a relatively large series of histopathological proved retroperitoneum GN cases, summarized the imaging features and further depicted risk factors of increased surgical blood loss.

**Methods:**

A total of 35 (18 male, 17 female) patients were retrospectively enrolled from January 2012 to June 2019 at our institution. Among them, 24 patients had undergone CT scans and 19 patients had undergone MR examination before treatment. The clinical and radiological features were analyzed and the relationships between image features and surgical blood loss were evaluated.

**Results:**

The media age of the involved 35 patients was 40 years (range, 14–66 years). The histological tumor size was 10.12 ± 4.56 cm for average. Retroperitoneum GN was relatively low density on unenhanced CT images and showed delayed progressive enhancement on enhanced CT and MR images. The whorled sign could be seen in 14 patients. The vessel encasement sign could be found in 17 patients. Univariate analysis revealed maximal tumor size measured on axial image, maximal tumor size measured on coronal image, encasing one or both renal pedicles, encasing the aorta and/or vena cava and whorled sign on MRI showed significant difference between the blood loss ≥ 400 ml and blood loss < 400 ml group. Logistic regression further detected that maximal tumor size measured on axial images (OR: 1.12; 95% CI: 1.02–1.24; *P* = 0.023) and encasing one or both renal pedicles (OR: 22.39; 95% CI: 1.35–372.99; *P* = 0.030) were independently correlated with surgical blood loss.

**Conclusions:**

Preoperative CT and MR imaging analysis was valuable for both diagnosis and surgical risk prediction of retroperitoneum GN.

## Background

Ganglioneuroma (GN) is a rare benign tumor which originates from neural crest cells. It is mainly composed of mature Schwann cells, ganglion cells and nerve fibers. GN may arise anywhere along the paravertebral sympathetic plexus. The retroperitoneum and posterior mediastinum are the two most common locations of GN [1–4]. Although GN is regarded as an uncommon tumor, more and more GNs have been detected with the dramatically increased use of computed tomography (CT) and magnetic resonance imaging (MRI). Though GNs are benign tumors, they may cause pain and compression symptoms and recurrence or malignant transformation has been reported [2, 5, 6]. Due to these characteristics, surgical resections and postoperative monitoring are recommended for GN patients.

There have been several studies describing the radiological appearances of GN [2, 7–16], but the reported case series are relatively small. To date, few studies have focused on the primary non-adrenal retroperitoneum GN. The precise diagnosis of non-adrenal retroperitoneum GN prior to surgery remains challenging. The non-invasive diagnosis of GN is important, because the surgical strategy and surgical approach can be different for benign and malignant tumors. Prior to the surgical procedure, CT and MR images can demonstrate important features of the tumors and help to narrow the differential diagnosis. Moreover, CT and MR images enable a multiplanar evaluation revealing the exact localization of the lesion and could heip to accurately evaluate the association of the mass with adjacent structure. Therefore, preoperative imaging of GNs could substantially facilitate preoperative planning. In this study, we reviewed a relatively large series of histopathological proved non-adrenal retroperitoneum GN cases at our institution and interpreted the CT and MRI features of them. Furthermore, we analyzed risk factors of increased surgical blood loss based on radiographic images for the first time.

## Methods

This study was a single-center retrospective study approved by the Committee on Ethics of Medicine of Changhai hospital and the study was carried out in accordance and relevant guidelines and regulations. The requirement to obtain written informed consent was waived by the Committee on Ethics of Medicine of Changhai hospital. Computerized patient record systems and the laboratory, pathology and radiology databases were searched from January 2012 to June 2019. The inclusion criteria were as follows: pathologically proven retroperitoneum GN; undergone contrast-enhanced CT or MRI before surgery. The exclusion criteria were: adrenal GN cases; absence of a presurgical contrast-enhanced CT or MRI record in our hospital; not undergoing surgical resection of tumors. Follow-up data were obtained by searching medical records in our hospital and making phone calls to the patients. The written informed consents were obtained from all patients.

### Patients

A total of 35 patients (18 male, 17 female) were pathologically diagnosed with retroperitoneum GN during the 7-years-period in our hospital. Their age ranged from 14 to 66 (median, 40 years). All of them were treated operatively. The mean time interval from preoperative imaging to surgery was 5 days (range, 0–27 days).

### Modalities

Among them, 24 patients had undergone CT scans and 19 patients had undergone MR examination before treatment. The CT scans were carried on by a 320-multidetector row CT system (Toshiba Medical Systems, Tokyo, Japan). The CT scan parameters were as follows: 120 kV, 150 effective mAs, a matrix of 350 × 350, and a gantry rotation time of 0.5 s. The intravenous iodinated contrast agent (Iopamiro, Bracco Sine Pharmaceuticals, Shanghai, China) was administered for contrast media enhancement. The contrast-enhanced CT scans were performed in arterial (20–25 s) and venous (60–70 s) phases after the contrast agent injection. MR imaging was performed by a 1.5 T MR system (GE Medical System, USA) with a body coil. The MR protocol contained horizontal T1 (repetition time (TR) 2.58 ms, echo time (TE) 1.18 ms) and T2 weighted (TR 6316 ms, TE 87 ms) images, coronal T2 weighted images (TR 7000 ms, TE 1230 ~ 1270 ms) and diffusion-weighted imaging (DWI) (TR 5000 ms, TE of 80 ms, b = 800). Images were obtained with a field of view of 440 × 440 mm, an image matrix of 224 × 270, and a slice thickness of 5 mm. Gadolinium-diethylenetriamine pantaacetic acid (Gd-DTPA, Magnevist, Berlin, Germany) was intravenously administrated for contrast media enhancement. The delayed time was 30 and 70 s after injection of contrast agent.

### Image analysis

**Fig. 1:**
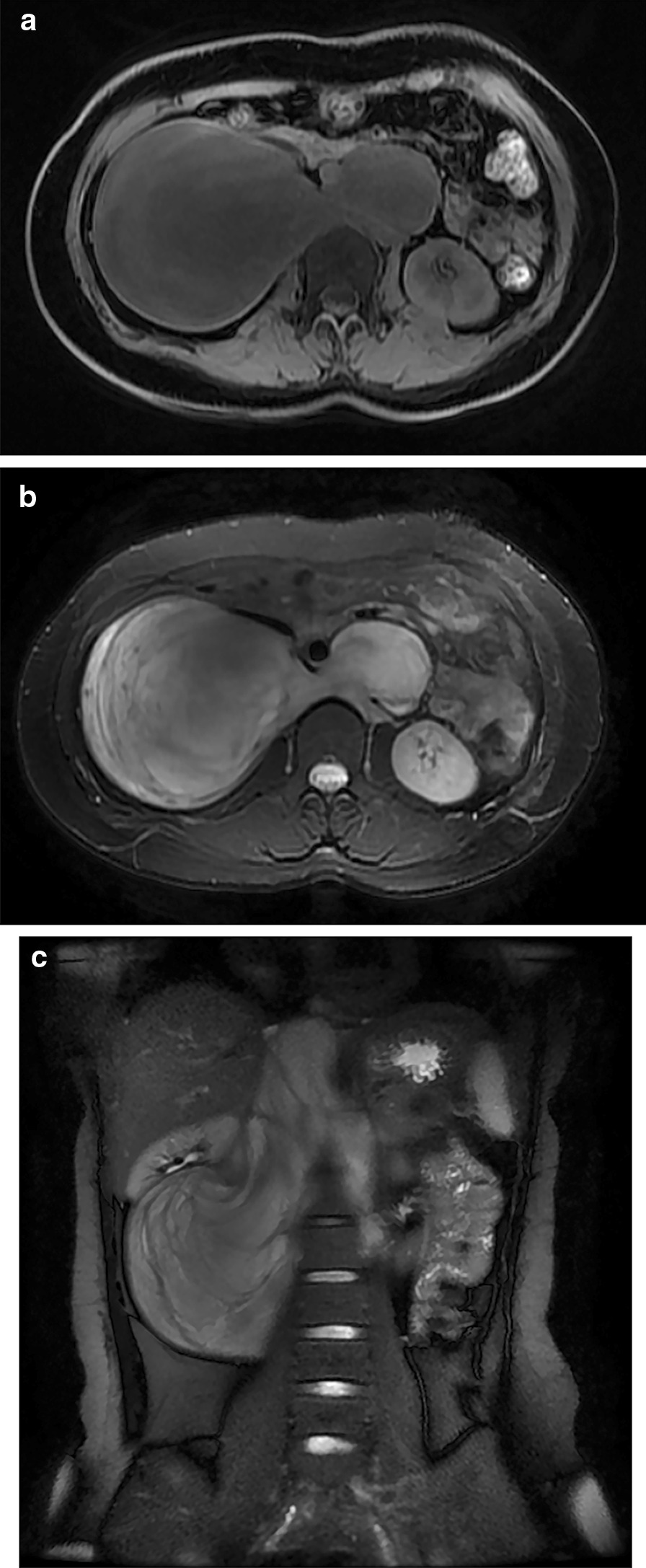
MR image of a 23-years-old female (patient no.25). The retroperitoneum ganglioneuroma was incidentally detected by a health check-up CT examination. **a** T1-weighted image. **b** axial T2-weighted image. c. coronal T2-weighted image. The whorled sign of retroperitoneum ganglioneuroma could be observed on T2-weighted image (**b**, **c**). The large amount of myxoid stroma presented predominantly high signal intensity on T2-weighted images and the cellular components including Schwann cells and ganglions may be responsible for the interlaced or nodular low signal intensity

The images were reviewed by two experienced radiologists (Zhang & Yang) independently, and discrepancies were resolved by consensus. The two readers were blinded to the operation records. The following radiological characteristics were assessed: tumor size (maximal diameter measured on axial and coronal 2D images), shape (round, oval or irregular), calcification (present or absent), density (CT)/ signal (MR) features, pre-contrast appearance (homogeneous or heterogeneous), the pattern of enhancement, specific signs including vessel encasement and whorled sign. The vessel encasement sign represented that the tumor showed the trend to surround major blood vessels (> 180°). The vessel encasement signs are categorized into 3 types: tumor encasing the origin of the coeliac axis, and/or of the superior mesenteric artery; tumor encasing one or both renal pedicles; tumor encasing the aorta and/or vena cava [17, 18]. The whorled sign was evaluated on MR T2-weighted images. The manifestation of the whorled sign was that the tumor was predominantly high intensity on T2-weighted image and an interlaced or nodular low-signal part can be seen within the tumor (Fig. 1). The whorled sign corresponded to the microscopic interlacing patterns of Schwann cells and collagen fibers within the tumor [10, 19]. All the clinical details, laboratory, pathological and follow-up data were also documented.

### Statistical analysis

The statistical analyses were performed with Predictive Analysis Software 18.0 (IBM, Armonk, New York, USA). Two-tailed *P* values were computed and a *P* value of less than 0.05 was considered to be statistically significant. All data were checked up with the Kolmogorov–Smirnov test to assess the normality. Continuous variables were showed as mean ± standard deviation (SD), while categorical variables presented as numbers or percentages. Differences between groups were assessed using the independent samples *t* test for continuous variables if they are conformed to the normal distribution and the Mann–Whitney U-test for those were not normally distributed. Logistic regression analysis was performed to examine associations between characteristics and surgical outcomes.

## Results

### Basic characteristics

Thirty-five retroperitoneum GN patients were enrolled in this study, including 18 male and 17 female patients. The baseline characteristics were displayed in Table 1, including age, gender, histological tumor size, symptoms, surgical blood loss, postoperative hospital stay. In detail, the mean age of the involved patients was 40 years, ranging from 14 to 66 years. The documented histological tumor size was 10.12 cm ± 4.56 cm for average. As for clinical manifestations, the tumor was asymptomatic in 25 patients (71.4% of the cases). The remaining 10 patients (28.6% of the cases) suffered from abdominal pain or discomfort (n = 7) and back pain (n = 3). Among the 25 asymptomatic patients, 21 cases were incidentally discovered by health check-ups, and 4 cases were detected when checking for other diseases such as cholecystitis, ureteral calculi and lumbar fracture. The adrenal hormonal levels of all the patients were within the normal range.

**Table 1:** Clinical and demographic characteristics of the 35 cases

Variables	No. of patients	Mean ± SD	Median (range)
*Gender*			
Male	18 (51.4%)		
Female	17 (48.6%)		
Age (years)		40.83 ± 12.96	40 (14–66)
*Symptoms*			
Incidentally detected	25 (71.4%)		
Abdominal pain or discomfort	7 (20.0%)		
Back pain	3 (8.6%)		
*Adrenal hormonal secretion*			
Normal	35(100%)		
Abnormal	0		
Histological tumor size (cm)		10.12 ± 4.56	9.5 (4–23)
*Surgical blood loss*			
≥ 400 ml	15 (42.9%)		
< 400 ml	20 (57.1%)		
Postoperative hospital stays (days)		7.03 ± 3.23	6 (3–18)

### Image findings

Twenty-four patients had undergone CT scans and 19 patients had undergone MR imaging. Eight patients had both CT and MR records. The imaging features were described in Tables 2 and 3 separately. In 17 cases, tumors tend to surround major blood vessels but without invasion or occlusion. None of the cases showed evidence of regional lymph node enlargement and visceral metastasis.

**Table 2:** CT findings in 24 patients with retroperitoneum ganglioneuroma

No	Age (year)	Gender	Tumor size (cm)		Shape	Calcification	Precontrast appearance	CT value (Hu)			Enhancement appearance	Vessel encasement	Boudary
			Axial	Coronal				Unenhanced	Arterial phase	Venous phase			
1	14	M	6.5	7.7	Oval	−	Homogeneous	36.5	38.1	39.9	Homogeneous	−	Well-defined
2	20	M	8.4	8.3	Lobulate	+	Homogeneous	27.3	27.5	28.1	Homogeneous	+	Well-defined
3	22	F	13.2	13.9	Lobulate	+	Heterogeneous	39.8	43.8	47.1	Heterogeneous	+	Well-defined
4	28	M	4.0	4.0	Oval	−	Homogeneous	40	41.8	42	Homogeneous	−	Well-defined
5	31	F	12.0	10.6	Lobulate	+	Homogeneous	29.4	30.9	38.6	Heterogeneous	−	Well-defined
6	32	M	2.2	3.1	Oval	−	Homogeneous	23.4	29.9	33.1	Homogeneous	−	Well-defined
7	33	M	9.5	13.4	Oval	+	Heterogeneous	19.4	25.1	27.4	Heterogeneous	+	Well-defined
8	35	M	8.4	13.5	Oval	+	Heterogeneous	30.7	46.3	67.1	Heterogeneous	−	Well-defined
9	36	F	2.8	2.6	Oval	−	Homogeneous	34.8	50.6	54.6	Homogeneous	−	Well-defined
10	37	M	5.7	8.8	Oval	−	Homogeneous	29.2	31.8	35.5	Homogeneous	−	Well-defined
11	38	M	13.4	16.7	Lobulate	−	Homogeneous	30.4	30.5	32.24	Homogeneous	+	Well-defined
12	39	M	3.7	4.2	Oval	−	Homogeneous	34.4	35	38.8	Homogeneous	+	Well-defined
13	40	F	16.2	13.8	Lobulate	+	Heterogeneous	42.3	47.6	53	Homogeneous	+	Well-defined
14	41	M	7.2	9.8	Lobulate	+	Homogeneous	27.4	29.1	31.7	Heterogeneous	+	Well-defined
15	44	F	4.9	6.7	Oval	−	Homogeneous	31.7	32.3	37.9	Heterogeneous	−	Well-defined
16	47	F	16.2	14	Lobulate	−	Heterogeneous	25.2	30.2	34.1	Heterogeneous	+	Well-defined
17	48	F	8.3	12	Lobulate	+	Heterogeneous	36.8	36	44.8	Heterogeneous	+	Well-defined
18	48	F	7.7	6.7	Oval	−	Homogeneous	33.6	31.8	39.9	Homogeneous	+	Well-defined
19	48	F	4.8	9.8	Lobulate	−	Homogeneous	34.2	40.1	45	Heterogeneous	+	Well-defined
20	52	M	3.7	4.1	Lobulate	−	Homogeneous	31.7	43.4	31.6	Homogeneous	−	Well-defined
21	56	F	4.8	5	Oval	−	Heterogeneous	22.4	40.1	73.6	Heterogeneous	+	Well-defined
22	57	F	6.6	6.7	Oval	−	Heterogeneous	53.2	71.5	86.9	Heterogeneous	+	Well-defined
23	63	F	3.3	4.9	Oval	−	Homogeneous	26.6	29.2	30.3	Homogeneous	+	Well-defined
24	66	M	5.7	8.0	Lobulate	−	Heterogeneous	30.3	34.5	35.1	Homogeneous	−	Well-defined

**Table 3:** MR findings in 19 patients with retroperitoneum ganglioneuroma

No	Age (year)	Gender	Tumor size (cm)		Shape	MR features			Whorled sign	Enhancement pattern	Vessle encasement	Boundary
			Axial	Coronal		T1WI	T2WI	DWI				
1	14	M	6.5	7.7	Oval	Hypointense	Heterogeneous high	Hyperintense	+	Homogeneous delayed	–	Well-circumscribed
2	20	M	8.4	8.3	Lobulate	Hypointense	Heterogeneous high	Hyperintense	+	Homogeneous delayed	+	Well-circumscribed
25	23	F	17.8	21.4	Irregular	Hypointense	Heterogeneous high	Hyperintense	+	Heterogeneous delayed	+	Well-circumscribed
26	27	M	8.2	10.3	Oval	Hypointense	Heterogeneous high	Hyperintense	−	Heterogeneous delayed	+	Well-circumscribed
27	29	F	8.7	6.6	Irregular	Hypointense	Heterogeneous high	Hyperintense	+	Heterogeneous delayed	−	Well-circumscribed
5	31	F	12.0	10.6	Lobulate	Hypointense	Heterogeneous high	Hyperintense	+	Heterogeneous delayed	−	Well-circumscribed
28	31	M	9.6	12	Oval	Hypointense	Heterogeneous high	Hyperintense	+	Heterogeneous delayed	−	Well-circumscribed
6	32	M	2.2	3.1	Oval	Hypointense	Homogeneous high	Hyperintense	−	Homogeneous delayed	−	Well-circumscribed
9	36	F	2.8	2.6	Oval	Hypointense	Heterogeneous high	Hyperintense	−	Homogeneous delayed	−	Well-circumscribed
29	36	M	8	6.2	Irregular	Hypointense	Heterogeneous high	Hyperintense	+	Homogeneous delayed	+	Well-circumscribed
14	41	M	7.2	9.8	Lobulate	Hypointense	Heterogeneous high	Hyperintense	+	Heterogeneous delayed	+	Well-circumscribed
30	42	M	5.2	4.7	Oval	Hypointense	Heterogeneous high	Isointense	+	Heterogeneous delayed	−	Well-circumscribed
15	44	F	4.9	6.7	Oval	Hypointense	Heterogeneous high	Isointense	+	Heterogeneous delayed	−	Well-circumscribed
31	44	F	7.7	10.2	Irregular	Hypointense	Heterogeneous high	Hyperintense	+	Heterogeneous delayed	−	Well-circumscribed
16	47	F	16.2	14	Lobulate	Hypointense	Heterogeneous high	Hyperintense	+	Heterogeneous delayed	+	Well-circumscribed
32	50	F	5.4	5.8	Oval	Hypointense	Heterogeneous high	Isointense	−	Heterogeneous delayed	−	Well-circumscribed
33	53	F	6.9	7.2	Irregular	Hypointense	Heterogeneous high	Hyperintense	+	Homogeneous delayed	−	Well-circumscribed
34	54	M	6.2	5.5	Oval	Hypointense	Homogeneous high	Hyperintense	−	No enhancement	−	Well-circumscribed
35	65	M	4.3	6.9	Oval	Hypointense	Heterogeneous high	Isointense	+	Heterogeneous delayed	−	Well-circumscribed

**Fig. 2:**
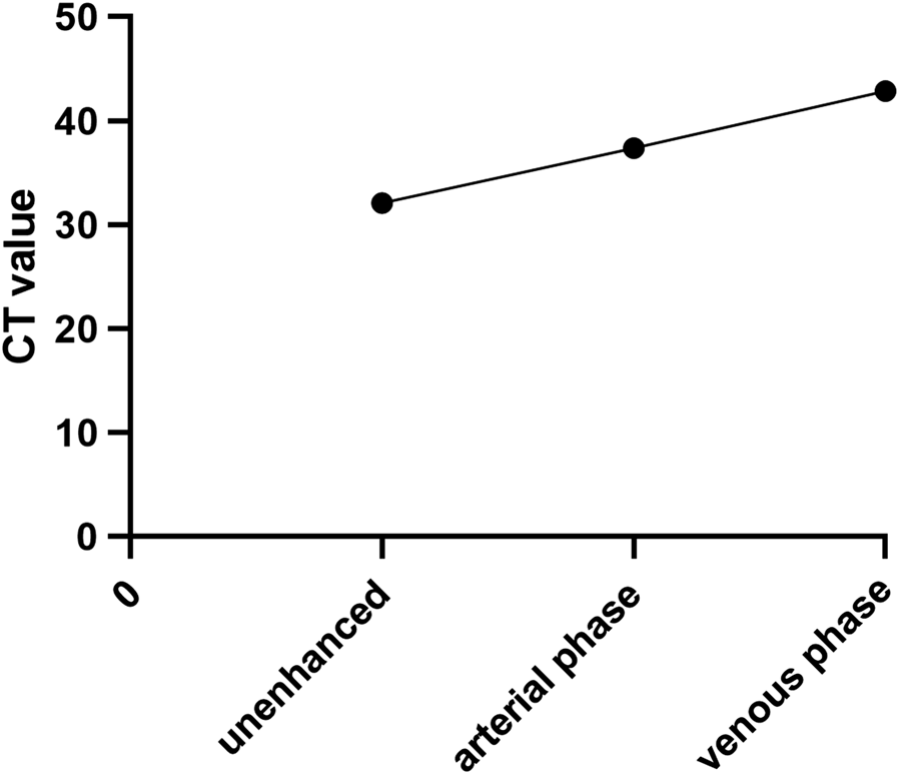
Enhancing pattern of ganglioneuroma on CT images. The progressive enhancement could be seen in typical ganglioneuroma

Twenty-four patients had undergone enhanced CT scans (Table 2). GNs presented hypodense on pre-contrast CT images, with a mean attenuation density of 32.11Hu (range, 19.4–53.2Hu). They were homogenous in 15 of 24 patients and heterogeneous in the remaining 9 patients. Punctate calcifications were found in 8 cases. After contrast, the progressive delayed enhancement was seen in 20 of 24 cases, while slightly contrast washout was observed in one case. In 3 cases, the tumor did not enhance at all. The enhancement pattern was shown in Fig. 2.

Nineteen patients had undergone enhanced MR imaging (Table 3). GNs revealed hypointense signals on T1-weighted images and heterogeneous hyperintense signals on T2-weighted images in all 19 patients. The whorled appearance was observed in 14 patients. GNs presented delayed heterogeneous enhancement in 12 patients and relatively homogenous enhancement in 6 patients. In one case, no enhancement was seen.

### Surgical findings and outcomes

Complete tumor resection was carried out in 34 cases (97.1%), and partial excision in 1 patient (2.9%) because the patient refused to take risks of nephrectomy. One patient underwent distal pancreatectomy and one underwent nephrectomy because of tumor adhesion. The median blood loss during surgery was 200 ml, ranging from 20 ml to 4000 ml. The estimated blood loss was over or equal to 400 ml in 15 cases and less than 400 ml in 20 cases. Severe surgical complications occurred in one patient (patient no.13) because of postoperative rebleeding that led to hypoperfusion. The patient died of multiple system organ failures on the 18th postoperative day. Follow-up data were available for 28 patients and the median follow-up duration was 22 months (range, 10–81 months). No recurrence or metastasis was found in these patients. Six patients were lost of follow-up because of the wrong phone number.

### Risk factors of increased blood loss during surgery

Maximal tumor size measured on axial image, maximal tumor size measured on coronal image, calcification, encasing one or both renal pedicles, encasing the aorta and/or vena cava and whorled sign on MRI showed significant difference between the blood loss ≥ 400 ml and blood loss < 400 ml group (Table 4A, B). Logistic regression revealed that maximal tumor size measured on axial images (OR: 1.12; 95% CI: 1.02–1.24; *P* = 0.023) and encasing one or both renal pedicles (OR: 22.39; 95% CI: 1.35–372.99; *P* = 0.030) were independently correlated with surgical blood loss (Table 5). Age, gender, encasing the origin of celiac axis, and/or of the superior mesenteric artery and CT value demonstrated no significant difference between the two groups.

**Table 4:** Comparison of Clinical and imaging parameters of GNs based on blood loss

Variables	Gender		Encasing the aorta and/or vena cava		Encasing one or both renal pedicles		Encasing the origin of CA and/or SMA		Calcification		Whorled sign	
	Male	Female	Yes	No	Yes	No	Yes	No	Yes	No	Yes	No
*A*												
Blood loss < 400 ml	12	8	4	16	2	18	1	19	1	12	6	5
Blood loss ≥ 400 ml	6	9	8	7	11	4	3	12	7	4	8	0
Fisher exact test	1.333		4.106		14.305		1.851		8.042		4.675	
*P* value (2-tailed)	0.248		0.043		< 0.01		0.174		0.005		0.031	

Variables	Age	Maximal diameter on axial images	Maximal diameter on coronal images	CT value		
				Unenhanced	Arterial phase	Venous phase
*B*						
Blood loss < 400 ml	40.50 ± 14.07	5.27 ± 1.82	6.46 ± 2.69	31.22 ± 5.02	37.93 ± 6.55	43.42 ± 13.59
Blood loss ≥ 400 ml	37.27 ± 10.74	10.77 ± 3.72	11.69 ± 4.09	33.16 ± 9.41	36.73 ± 13.40	42.17 ± 12.90
*t* value	1.430	− 5.274	− 4.599	− 0.645	0.287	0.202
*P* value (2-tailed)	0.162	< 0.01	< 0.01	0.526	0.777	0.842

SMA, superior mesenteric artery; CA, the coeliac axis

**Table 5:** Logistic regression analysis on the risk factors of blood loss ≥ 400 ml during surgery

Variables	OR (95%CI)	*P* value
Maximal diameter on axial images	1.12 (1.02–1.24)	0.023
Encasing one or both renal pedicles	22.39 (1.35–372.99)	0.030

## Discussion

To our knowledge, this study is the largest case series of retroperitoneum GNs and this was the first study evaluating imaging-based risk factors of increased surgical blood loss. In this case series, retroperitoneum GNs were recognized to be hormonally inactive, mostly incidentally discovered and with satisfying outcomes after surgery. Since GNs are mostly hormone-silent and asymptomatic and retroperitoneum space is deep and extensive, they can be huge when detected. These findings were consistent with previous studies [1, 4, 20]. The gender incidence of GN varies in literature [7, 8, 14, 21, 22]. In our experience, we found a similar GN morbidity between females and males.

Retroperitoneum GNs in our study displayed as well-defined hypodense masses on unenhanced CT images, mostly homogenous. Punctate calcifications were found in 33.3% cases. The sight or moderate progressive enhancement was observed in most patients. On MR images, GNs often presented to be hypointense on T1-weighted images, heterogeneous hyperintense on T2-weighted images and hyperintense on DWI images. These imaging features were similar to previous literature reports [1, 3, 4, 8, 9, 11, 13, 14, 19, 23]. The whorled sign, as previously reported, was considered as a specific sign of GN [9, 10, 19]. In our study, the whorled sign could be seen in 73.7% patients and it was in accordance with the pathological components of GN. The large amount of myxoid stroma presented predominantly high signal intensity on T2-weighted images. The cellular components, including Schwann cells and ganglions, may be responsible for the interlaced or nodular low signal intensity [1, 3, 7]. Interestingly, retroperitoneum GNs tend to extend along the inter-vessel space and partially or wholly surround major blood vessels, with nearly half of the patients (17/35) presented with vessel encasement signs. Under this circumstance, the GNs could be confused with malignant tumors.

The differential diagnosis of GN includes many malignant and benign tumors, including neuroblastoma (NB), ganglioneuroblastoma (GNB), schwannoma, lymphangioma and so on. NB, GNB and GN originate from the ganglion cells. The three tumors differ in their degree of cellular and extracellular maturation. NB are highly malignant lesions and GNB has intermediate malignant potential. NB are more aggressive and occur in younger patients (median age, under 2 years). NB and GNB often present as irregularly shaped, unencapsulated lesions and tend to be inhomogeneous owing to tumor necrosis and hemorrhage. They have the trend to invade adjacent organs and adjacent vessels and tend to metastasize to bone, bone marrow, liver and lymph nodes [1]. Regional invasion of paraspinal musculature and psoas may occur, and invasion of the neural foramen into the epidural space is also frequent. In our retroperitoneum GN cases, no invading of the vessels and organs was observed, which suggested benign tumors. Moreover, metastasis was rarely seen in GNs. Schwannomas arise from the nerve sheath and constitute the majority of neurogenic retroperitoneum neoplasms. They are commonly diagnosed in patients in the 2nd to 5th decades of life. Schwannomas are composed of alternating Antony A and Antony B areas and degenerative parts [24]. Schwannomas are encapsulated masses and extend along the nerve course. Large schwannomas may present prominent cystic degeneration and calcification. Schwannomas have been reported to exhibit a target-like appearance, i.e., peripheral high-signal intensity and central low-signal intensity, on T2 weighted images [25]. Retroperitoneal lymphangiomas can present at any age and are common in males. They often present as unilocular or multilocular thin-walled cysts with no enhancement.

**Fig. 3:**
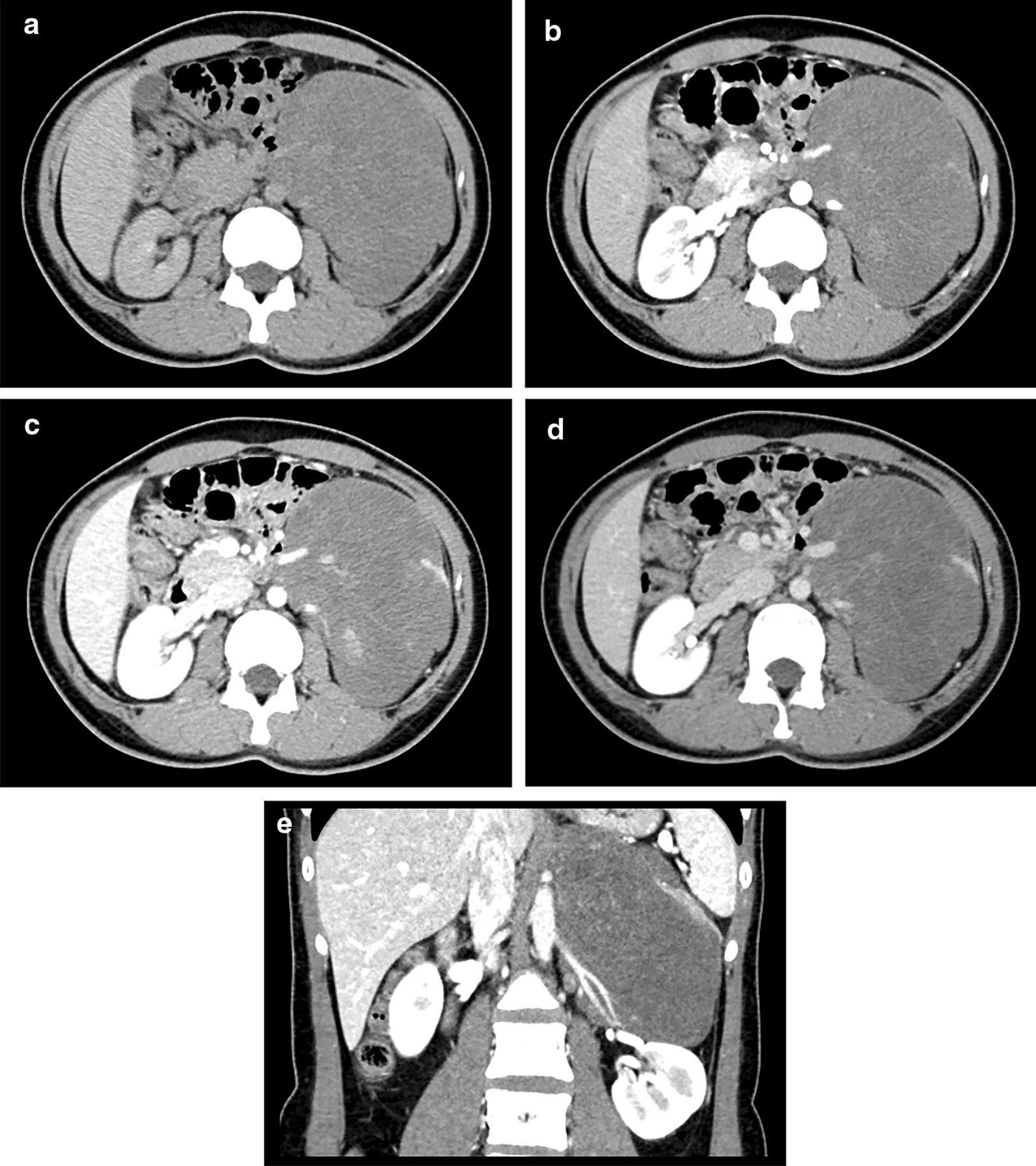
CT image of a 22-years-old female (patient no.3). The retroperitoneum ganglioneuroma was incidentally detected by the health examination after college graduation. **a** Non-enhanced image. **b** arterial phase. **c** portal phase. **d** delayed phase. **e** coronal image. The tumor encased the left renal pedicle, and the patient lost 900 ml blood during surgery

Surgical treatment is the optimal choice for the treatment of retroperitoneum GNs [2, 26]. According to the experience of our institution, complete rection was possible for most patients. However, since GNs may adhere to tissues nearby and surround the major adjacent vascular structures [12], the resection can be complex and surgical risks should not be underestimated. In this study, the estimated blood loss was over or equal to 400 ml in 15 cases during the operation, with the maximum blood loss as 4000 ml. A large amount of bleeding may lead to blood transfusion, even to organ ischemia–reperfusion injury, shock and death. As a result, preoperative radiological interpretations of retroperitoneum GNs are crucial. Importantly, we have found that larger tumor size measured on axial images and encasing one or both renal pedicles (Fig. 3) may lead to increased blood loss during surgery. Moreover, we have found that the maximal diameters measured on coronal 2D images of retroperitoneum GNs were more extensive than those measured on axial images in 68.6% (24/35) cases, which was in accordance with the potential of GNs to grow vertically from the sympathetic ganglia [2]. These findings may help the surgeon to predict surgical risk, select surgical technique, and carry out surgical treatment. Our follow-up data, consistent with previous studies [15, 16], indicated the good prognosis of retroperitoneum GNs after surgical procedure.

We acknowledged that our study had several limitations. First, this was a single-center retrospective study, which may lead to the bias of data. Also, those patients who did not receive surgery were not enrolled, which could limit the representation of our case series. Despite these limitations, we believe that our data indeed provided valuable information on retroperitoneum GN. And studies with large sample sizes will be carried out for further verification.

## Conclusions

In summary, CT and MR displayed vital roles in the diagnosis and pre-operative evaluation of GNs. Though GNs are uncommon tumors, they should be taken into consideration when specific features are presented. These features are as follows: (a) relatively low density on unenhanced CT images, (b) the whorled sign on T2-weighted images, (c) the possible tendency to surround major vessels but with no narrowing, (d) the delayed progressive enhancement on enhanced CT and MR images. Preoperative images could provide image-defined risk factors for surgery. Larger tumor size measured on axial images and encasing one or both renal pedicles may lead to increased blood loss during surgery.

## Data Availability

Not applicable.

## Abbreviations

GN: Ganglioneuroma
CT: Computed tomography
MRI: Magnetic resonance imaging
SD: Standard deviation
SMA: Superior mesenteric artery
CA: The celiac axis
NB: Neuroblastoma
GNB: Ganglioneuroblastoma
